# Brazilian Adaptation of the Coronavirus Anxiety Scale: A Psychometric Investigation of a Measure of Coronaphobia

**DOI:** 10.1177/0030222821991325

**Published:** 2021-02-02

**Authors:** Fernando E. Padovan-Neto, Sherman A. Lee, Rayanne Poletti Guimarães, Lívea Dornela Godoy, Hugo Bononi Costa, Francisco Luiz Silva Zerbini, Sérgio S. Fukusima

**Affiliations:** 1 Department of Psychology, Faculty of Philosophy, Sciences and Letters of Ribeirão Preto, University of São Paulo, São Paulo, Brazil; 2 Department of Psychology, Christopher Newport University, Newport News, Virginia, United States; 3 Ribeirão Preto Medical School, University of São Paulo, São Paulo, Brazil

**Keywords:** coronavirus, COVID-19, anxiety, Brazil, coronaphobia

## Abstract

This study examined the psychometric properties of a Brazilian adapted version of the Coronavirus Anxiety Scale (CAS-BR) in a sample of adults in Brazil. Confirmatory factor analyses demonstrated that the CAS-BR produces a reliable (α = .84), unidimensional construct whose structure was shown to be invariant across gender, race, and age. However, some items of the CAS-BR were stronger indicators of the coronavirus anxiety construct for women and younger adults. Although the CAS-BR demonstrated evidence of discrimination ability for functional impairment (AUC = .77), Youden indexes were low to identify a clinical cut-score. Construct validity was demonstrated with correlations between CAS-BR scores and measures of functional impairment, generalized anxiety, and depression. Exploratory analyses revealed that CAS-BR total scores were higher among women and participants with a history of anxiety disorder. These findings are consistent with previous investigations and support the validity of CAS-BR for measuring coronavirus anxiety with Brazilian adults.

A novel viral pneumonia originating from China was announced to the World Health Organization on December 31, 2019 (WHO, 2020). In a just a few months this infectious disease caused an unprecedented number of deaths, disruptions, and economic hardships all around the world. Not surprisingly, many people’s mental health began to suffer as a consequence of this pandemic. For example, surveys from around the globe reported elevated levels of generalized anxiety and depression among the people during the pandemic (Mazza et al., 2020; Sønderskov et al., 2020; Wang et al., 2020; Xie et al., 2020). Alcohol use also increased during this time, which may have led to or worsened many people’s stresses and existing mental health problems (Pollard et al., 2020). One psychological factor that appeared to be contributing to this pattern of pandemic distress has been coronavirus anxiety.

A significant proportion of the world’s population have experienced fear and anxiety over the coronavirus because of its highly contagious, mysterious, and potentially lethal qualities. For instance, more than 80% of the adults surveyed in India were worried about the coronavirus (Roy et al., 2020), while the rate was lower, but still high at 66.5% for American adults (Bycoffe et al., 2020). Because there continues to be no proven cure or vaccine for this virus, the notion of being infected or infecting loved ones with the coronavirus is especially frightening. And although it is natural and safe to be weary of any infectious disease, high levels of fear and anxiety over the coronavirus have been problematic. In fact, this particular condition has been tied to unhealthy behaviors, such as drug and alcohol coping, as well as deep psychological issues, such as extreme hopelessness, suicidal ideation, and disorientation of core beliefs ( Lee et al., 2020b; Lee et al., 2020a; Milman et al., 2020). Moreover, coronavirus anxiety has been shown to predict generalized anxiety, depression, and functional impairments, beyond personality traits and COVID-19 factors (Lee & Crunk, 2020; Lee et al., 2020b; Nikčevića & Spada, 2020). Therefore, research into this form of pandemic psychopathology is extremely important as COVID-19 continues to wear down the mental health and well-being of people around the world.

Coronavirus anxiety appears to be a global phenomenon. And although the Coronavirus Anxiety Scale (CAS; Lee, 2020), which is an empirically supported mental health screener of this form of pandemic psychopathology, has been adapted for use in such countries as Bangladesh (Ahmed et al., 2020), Turkey (Evren et al., 2020), Peru (Franco-Jimenez, 2020), and Mexico (Mora-Maganã et al., 2020), there has been no known validation research of this kind in Brazil. This is surprising given that as of November 21, 2020 Brazil held the 3rd largest number of confirmed cases of COVID-19 in the world at 5,981,767 and the 2nd largest rate of confirmed deaths from this disease at 168,061 deaths (793.65/million population) (WHO, 2020). The COVID-19 pandemic triggered a series of problems in Brazil that might have impacted negatively in Brazilians’ mental health. Recent data has shown that during the pandemic frequent feelings of sadness/depression affected 40% of adult Brazilians, and frequent sensation of anxiety and nervousness was reported by more than 50% of them (Barros et al., 2020). Not only the virus itself but fake news, government crisis, health insecurity, economy collapse, and conflict between science and the State policies have also played an important role in building a general sense fear and anxiety. Brazil has the world’s highest prevalence of anxiety (WHO, 2017). Therefore, examining the validity of the coronaphobia construct in Brazilian adults is relevant since there is an urgent need to adapt a reliable and executable instrument to assess COVID-19-related anxiety in Brazil. Thus, the purpose of this study was to examine the psychometric properties of a Brazilian adaptation of the CAS (CAS-BR) using a sample of adults residing in Brazil.

In this study, we first began the research by creating a Brazilian adapted version of the CAS using a standard translation process. Second, we collected data using an online survey with the CAS-BR and other related measures. Third, we analyzed the factorial validity of the CAS-BR using a confirmatory factor analysis (CFA) of the five items of the measure. Fourth, we examined the CAS-BR for measurement invariance across age, gender, and race, using multiple groups CFAs. Fifth, we employed Receiver Operating Characteristic (ROC) analyses to test the classification features of the CAS-BR to correctly identify individuals who experienced clinically significant anxiety. Sixth, we examined the construct validity of CAS-BR by examining its correlations with measures of depression, generalized anxiety, and functional impairment. Last, we explored demographic differences in mean-levels of CAS-BR scores to identify vulnerable groups.

## Method

### Participants and Procedure

Anonymous online survey collected data from 505 Brazilian adults between July 31 and September 13, 2020. During this period, the number of cases increased from 2,552,265 to 3,109,630. The number of deaths raised from 90,134 to 103,026 (WHO, 2020). Participants were recruited using social media platforms (e.g. Facebook, WhatsApp, and Instagram) and were asked to volunteer in this research project without compensation. Prior to providing informed consent, the participants were given details regarding the nature, objectives, risks/benefits, and anonymity of responses for this study.

The study’s sample consisted of 305 women, 194 men, and 6 other gender. Most of the participants earned a Bachelor’s degree or higher (*n* = 348; 68.9%) and had a combined median age of 32 (18–77) years. Most of the participants identified as White (*n* = 387; 76.6%), followed by Brown (*n* = 75; 14.9%), Black (*n* = 20; 4.0%), Yellow (*n* = 12; 2.4%), not reported (*n* = 6; 1.2%) and other race (*n* = 5; 1.0%). Most of the participants were married (*n* = 199; 39.4%), followed by single (*n* = 194; 38.4%) and other marital status (*n* = 112; 22.2%). Most of the participants were from the Southeast (*n* = 367; 72.7%), followed by the South (*n* = 68; 13.5%), Midwest (*n* = 38; 7.5%), Northeast (*n* = 22; 4.4%), and North (*n* = 10; 2.0%) regions of Brazil. The majority of the participants had not been diagnosed with COVID-19 (*n* = 482; 95.4%), did not have contact with others who have been diagnosed with COVID-19 (*n* = 306; 60.6%), did not have a friend or family member die from COVID-19 (*n* = 324; 64.2%), and have never been diagnosed with an anxiety disorder (*n* = 338; 66.9%).

### Measures

The CAS-BR and Working and Social Adjustment Scale (WSAS) were translated from English to Brazilian Portuguese. All of the psychological scales used in this study were already validated. Specifically, we used Moreno et al.’s (2016) Brazilian adapted scale for our measure of generalized anxiety and Santos et al.’s (2013) Brazilian adapted scale for our measure of depression. We translated the measures of CAS-BR and WSAS using established guidelines (Beaton et al., 2000; Reichenheim & Moraes, 2007). First, we used two bilingual experts to translate these measures from English to Brazilian Portuguese. Next, two Brazilian psychiatrists with expertise in anxiety disorders reviewed and made slight adjustments to the language of the measures to improve their readability. These adjustments were meant to focus on functional rather than on literal equivalence. These versions of the measures were then back translated into English by two independent Brazilian back-translators who did not participate in the first translation step. Next, the researchers of this study compared the original (English) and the back translated (Brazilian Portuguese) measures with one another and judged them to be functionally equivalent. A pilot test of these finalized measures using a group of eight Brazilian adults demonstrated that all of them understood each item of the measures perfectly and without problems.

#### Background Information

Participants were asked to report their age, gender, race, marital status, education, area of residence, COVID-19 diagnosis, contact with anyone with COVID-19, knowledge of a friend or family member who died from COVID-19, and history of anxiety disorder.

#### Psychological Distress

Adapted versions of the Generalized Anxiety Disorder scale (GAD-7; Spitzer et al., 2006) and Patient Health Questionnaire (PHQ-9; Kroenke et al., 2001) were used to measure clinical symptoms of generalized anxiety and depression, respectively. Using a 4-point time anchored scale (0 = *not at all* to 3 = *nearly every day*), participants rated how frequently they experienced symptoms of generalized anxiety (e.g., “Trouble relaxing.”) and depression (e.g., “Feeling tired or having no energy.”) over the previous two weeks. In previous studies, the Brazilian Portuguese version of the PHQ‐9 presented excellent indicator in respect to validity and reliability (Moreno et al., 2016). The PHQ-9 also demonstrated validity (de Lima Osorio et al., 2009; Santos et al., 2013) and adequate reliability (Bergerot et al., 2014) in studies conducted in Brazil. In this study, both adapted versions of the GAD-7 (α = .93) and PHQ-9 (α = .92) exhibited excellent levels of internal consistency reliability.

##### Functional Impairment

An adapted version of Mundt et al.’s (2002) Work and Social Adjustment Scale (WSAS) was used to measure functional impairment . Participants were asked to rate five items of the WSAS, using a 9-point severity scale (0 = *not at all* to 8 = *very severely*), regarding how much impairment they experienced due to fear and anxiety over the coronavirus (e.g., “Because of my fear and anxiety over the coronavirus, my ability to work is impaired.”). Based on a WSAS cut-score of ≥21, 38.6% of the sample experienced functional impairment. This adapted scale exhibited fair internal consistency reliability (α = .79), within the conditions of use in accordance to the original study (Ferreira et al., 2018).

##### Social Attitudes

Participants were asked to rate, using a 5-point scale (1 = very dissatisfied to 5 = very satisfied), their satisfaction with Brazilian Government (*M* = 1.74; *SD* = 1.06) by the item, “Overall, how satisfied are you with Government's responses to COVID-19?”

##### Coronavirus Anxiety

An adapted version (CAS-BR) of the Coronavirus Anxiety Scale (CAS; Lee, 2020) was used to measure coronavirus related fear and anxiety (see Table 1). The CAS-BR items measure physiologically-based symptoms that are experienced when participants are triggered by coronavirus-related information and thoughts (e.g., “I felt dizzy, lightheaded, or faint, when I read or listened to news about the coronavirus.”). Using a 5-point time anchored scale (0 = *not at all* to 4 = *nearly every day over the last 2 weeks*), participants rated how frequently they experienced each anxiety symptom. The CAS-BR exhibited good internal consistency reliability (α = .84).

**Table 1.** Coronavirus Anxiety Scale—Brazilian Portuguese Version (CAS-BR).

CAS-BR						
Com que frequência você experimentou as seguintes situações nas últimas 2 semanas?		*Nunca*	*Raro, menos que um ou dois dias*	*Vários dias*	*Mais de 7 dias*	*Quase todos os dias durante as 2 últimas semanas*
1.	Senti tonturas, fiquei desorientado (com a sensação de não saber onde eu estava) ou tive sensação de desmaio quando li ou ouvi notícias sobre o coronavírus.	0	1	2	3	4
2.	Tive dificuldade em adormecer ou manter o sono porque estava pensando no coronavírus.	0	1	2	3	4
3.	Senti-me paralisado ou “em choque” quando pensei ou fui exposto a informações sobre o coronavírus.	0	1	2	3	4
4.	Perdi o apetite quando pensei ou fui exposto a informações sobre o coronavírus.	0	1	2	3	4
5.	Senti náuseas ou tive problemas de estômago quando pensei ou fui exposto a informações sobre o coronavírus.	0	1	2	3	4
	Totais das colunas	_____ +	_____ +	_____ +	_____ +	_____ +
					Pontuação Total **________**	

*Note*. The CAS-BR is placed in the public domain to encourage its use in clinical assessment and research. No formal permission is therefore required for its reproduction and use by others, beyond appropriate citation of the present article.

### Statistical Approach

A series of bootstrap (2000 samples) maximum likelihood confirmatory factor analyses (CFAs) were run on the CAS-BR to test the instrument’s factor structure and invariance across demographic groups. Conventional standards were used to determine goodness of fit and invariance (Brown, 2006; Byrne, 2001). Specifically, acceptable fit for a CFA model was defined by a standardized root-mean-square residual (SRMR) value ≤0.05, root-mean-square-error of approximation (RMSEA) value ≤0.10, and comparative fit index (CFI) and Tucker Lewis index (TLI) values ≥0.90. Invariance was defined by acceptable model fit statistics as well as a non-significant value (*p* ≥ .05) on a chi-square difference test.

A receiver operating characteristic (ROC) analysis was used to exam the classification features of the CAS-BR and identify the optimal cut-score for psychiatric screening purposes. Based on the results of the original CAS study (Lee, 2020), properties of similar types of psychiatric screening tests (Spitzer et al., 2006; van Dam et al., 2013; Weinstein et al., 1989) and diagnostic criteria (Schisterman et al., 2005; Šimundić, 2009), the following guidelines were used: (1) area under the curve (AUC) value ≥.70, (2) a convex shaped ROC curve, and (3) the optimal cut-score should have a sensitivity value ≥80%, specificity value ≥70%, and yield the highest Youden index among scores above .50.

Zero-order correlations were run between the scores of the CAS-BR and the measures of functional impairment, generalized anxiety, and depression to examine the construct validity of the CAS-BR. Because coronavirus anxiety scores have been shown in previous research to correlate positively with these measures of psychological distress and disability (Lee, 2020; Leet et al., 2020a; Nikčevića & Spada, 2020), we expected to find a similar pattern of results using this sample of Brazilian adults. Independent samples *t*-tests were run to explore differences in mean level CAS-BR scores between demographic groups. All of the statistical analyses were calculated using SPSS version 26.0, except for the CFAs, which were run using AMOS version 25.0.

## Results

### Confirmatory Factor Analyses

A preliminary screening of the data suggested that the CAS-BR items were suitable for factor analysis (Tabachnick et al., 2007). Specifically, the data did not exhibit problems pertaining to sample size, missing data, nonnormality, multicollinearity, or singularity. Moreover, the correlation matrices were deemed factorable (Bartlett’s test of sphericity = *p* < .001; Kaiser-Meyer-Olkin test = .84).

**Figure 1.**
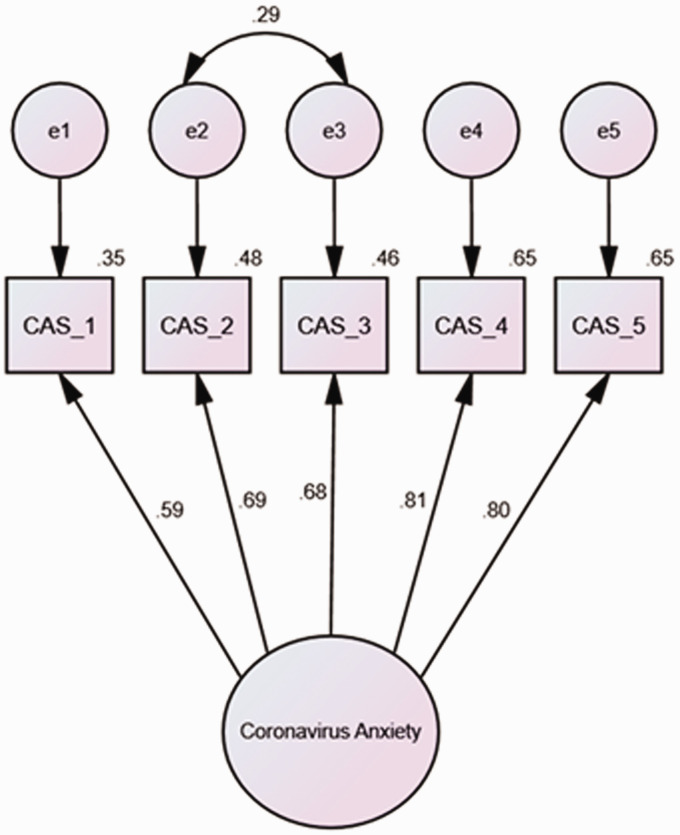
Model Based on Bootstrap Maximum Likelihood (ML) Estimations (2000 Samples). *Note*. All of the standardized coefficients are significant at the .05 level. CAS_1 (item #1) = Dizziness; CAS_2 (item #2) = Sleep Disturbance; CAS_3 (item #3) = Paralysis; CAS_4 (item #4) = appetite loss; CAS_5 (item #5) = Digestive Distress.

A CFA was run to test whether or not the items for this adapted version of the CAS (Lee, 2020) cohered together into a single, coronavirus anxiety construct using a sample of adults from Brazil. The results showed that the single-factor model was internally consistent (α = .84) and yielded acceptable model fit [χ^2^(5) = 42.89, *p* < .001] for all of the indices [CFI = .96; TLI = .92; SRMR = .02], except one [RMSEA = .12, 90% CI (.09, .16)]. Examination of the modification indices and standardized residuals identified error terms that contributed to model misfit. Specifically, the CAS-BR items 2 (sleep) and 3 (paralysis) were thematically similar because they both have to do with states of immobility. When these error terms were allowed to correlate (see Figure 1), the model yielded excellent fit [χ^2^(4) = 14.10, *p* < .01] for all of the indices [CFI = .99; TLI = .97; SRMR = .02; RMSEA = .07, 90% CI (.03, .11)]. Therefore, the CAS-BR demonstrated factorial validity.

Multigroup CFAs were run to examine if the coronavirus anxiety construct was being measured the same way across the demographic variables of gender (women vs men and other), age (18 to 29 vs 30 and older), and race (Whites vs non-Whites). The results demonstrated that there was a gender difference between females and non-females. Although there was excellent model fit [χ^2^(8) = 31.70, *p* < .001] for all of the indices [CFI = .98; TLI = .94; SRMR = .03; RMSEA = .08 (.05,.11; 90% CI)], indicating configural invariance, a significant increase in χ^2^ value [Δχ^2^ (5) = 34.80, *p* < .001] between the models indicated that the model did not pass the test for metric invariance. Analysis of the regression weights showed that items #2 (sleep) and #3 (paralysis) were stronger indicators of coronavirus anxiety for females than the non-females.

The results demonstrated that there was also an age difference between older (30 years and older) and younger (18 to 29 years old) adults. Although there was excellent model fit [χ^2^(10) = 54.6, *p* < .001] for all of the indices [CFI = .96; TLI = .91; SRMR = .05; RMSEA = .09 (.07,.12; 90% CI)], indicating configural invariance, a significant increase in χ^2^ value [Δχ^2^ (5) = 13.1, *p* = 0.02] between the models indicated that the model did not pass the test for metric invariance. Analysis of the regression weights showed that item #4 (appetite loss) was a stronger indicator of coronavirus anxiety for younger adults than the older adults. However, the results demonstrated no race differences between Whites and non-Whites, which were evidenced by excellent model fit [χ^2^(10) = 28.31, *p* < .01] for all of the indices [CFI = .96; TLI = .91; SRMR = .04; RMSEA = .09 (.07,.12; 90% CI)] and a non-significant increase in χ^2^ value [Δχ^2^ (5) = 9.4, *p* = 0.09, *ns*] between the models. Overall, these findings showed that the coronavirus anxiety construct was measured adequately across demographic groups (i.e., configural invariance). However, there were notable differences in terms of some items of the CAS-BR being stronger indicators of the coronavirus anxiety construct for women and younger adults compared to their counterparts. The only demographic variable where the measurement of the coronavirus anxiety construct was indistinguishable between groups was race.

### Receiver Operating Characteristic Analyses

Receiver operating characteristic (ROC) analyses were used to evaluate the diagnostic viability of the CAS as a mental health screening tool, as well as determine a cut score that best distinguishes individuals who experience clinically significant impairment because of coronavirus anxiety (individuals who scored > 20 on the WSAS) from those who were not impaired by this form of anxiety. Although a ROC graph displayed the convex pattern that is indicative of good discrimination ability and the area under the curve (AUC) demonstrated fair diagnostic accuracy for the CAS-BR (AUC =.77 *p* < .001), Youden indices were all too low (i.e., < .50) to identify a meaningful cut-point to classifying individuals with functional impairment due to coronavirus anxiety. Therefore, we were unable to identify an optimal cut-score for this Brazilian adapted version of the CAS.

#### Independent Samples t-Tests and Correlations

Independent samples *t*-tests were used to explore mean level differences between demographic groups. The results revealed that coronavirus anxiety was higher among women (*M* = 3.28; *SD* = 3.59) compared to the non-women (*M* = 1.71; *SD* = 2.47), *t*(501.78) = 5.81, *p* < .001, participants with a history of anxiety disorder (*M* = 3.98; *SD* = 4.06) compared to participants without a history of anxiety disorder (*M* = 2.00; *SD* = 2.59), *t*(234.61) = 5.75, *p* < .001, as well as, participants very dissatisfied or dissatisfied with Government’s responses to COVID-19 (*M* = 2.87; *SD* = 3.36) compared to participants that were indifferent, satisfied or very satisfied with Brazilian Government (*M* = 1.55; *SD* = 2.61), *t*(503) = 3.37, *p* < .001. However, coronavirus anxiety levels were not different between Whites (*M* = 2.54; *SD* = 3.14) and non-Whites (*M* = 3.04; *SD* = 3.70), *t*(503) = 1.34, *p* = .15, *ns*., married participants (*M* = 2.34; *SD* = 3.08) and non-married participants (*M* = 2.86; *SD* = 3.40), *t*(503) = −1.74, *p* = .08, *ns*., older participants (*M* = 2.46; *SD* = 3.18) and younger participants (*M* = 2.96; *SD* = 3.42), *t*(503) = −1.65, *p* = .10, *ns*., participants living in the Southeast region of Brazil (*M* = 2.66; *SD* = 3.31) and participants residing outside of the Southeast region of Brazil (*M* = 2.64; *SD* = 3.24), *t*(503) = 1.05, *p* = .96, *ns*., participants educated at the Bachelor’s level and above (*M* = 2.55; *SD* = 3.18) and participants educated below a Bachelor’s level (*M* = 2.90; *SD* = 3.51), *t*(503) = −1.14, *p* = .26, *ns*., participants who had contact with people diagnosed with COVID-19 (*M* = 2.72; *SD* = 3.41) and participants who did not have contact with people diagnosed with COVID-19 (*M* = 2.61; *SD* = 3.21), *t*(503) = .37, *p* = .72, *ns*., and participants who had family or a close friend die from COVID-19 (*M* = 2.94; *SD* = 3.54) and participants who did not have family or a close friend die from COVID-19 (*M* = 2.50; *SD* = 3.12), *t*(503) = 1.47, *p* = .14, *ns*. Overall, gender, history of anxiety disorder, and participants’ dissatisfaction with Government’s responses to COVID-19 were the only demographic variables to demonstrate mean level differences in CAS-BR scores.

Bivariate correlation analyses were used to examine the relationship between the CAS-BR scores and measures of functional impairment and psychological distress. The results showed that CAS-BR were shown to be positively correlated with scores from measures of functional impairment (*r* = .55, *p* < .001), generalized anxiety (*r* = .57, *p* < .001), and depression (*r* = .54, *p* < .001). Overall, these patterns confirmed our expectations and provide construct validity evidence for the CAS-BR.

## Discussion

The purpose of this study was to examine the psychometric properties of the Brazilian adapted version of the CAS (Lee, 2020) in a sample of 505 adults. Our results confirm previous findings of the original investigation of the CAS, and support the validity of CAS-BR for measuring coronavirus anxiety with Brazilian adults. The data presented here can help to identify people particularly emotionally affected by the pandemic, as well as, to assess the effectiveness of intervention programs for the management of anxiety.

The COVID-19 pandemic had a negative impact on the mental health of Brazilians. According to recent studies, the most affected groups were young adults, women, participants with a previous diagnosis of mental disorder and individuals at higher risk from coronavirus (Duarte et al., 2020; Goularte et al., 2021; Seco Ferreira et al., 2020). Anxiety, depression and sleep disorders were the most common psychiatric symptoms in essential workers (De Boni et al., 2020) and health professionals (Barroso et al., 2020). Also, the lifestyle changes imposed by the pandemic decreased the practice of physical activities and increased the consumption of alcohol and tobacco. Data collected by the Oswaldo Cruz Foundation (https://convid.fiocruz.br/) showed that there is a positive correlation between the symptoms of anxiety and depression with the consumption of these substances. In addition, sedentarism and physical inactivity were associated with worse results in screening tools for mental disorders (Werneck et al., 2020).

So far, there are no parameters to estimate the psychological and psychiatric impact that the new coronavirus pandemic has had on the Brazilian population. Public health actions such as social distancing are extremely necessary to reduce the spread of COVID-19. However, these actions can increase anxiety. Therefore, it is essential to implement public mental health policies during and after the pandemic (Ornell et al., 2020).

The CAS-BR is an important psychometric instrument to measure coronaphobia. Mental health professionals can use the CAS-BR as a valuable tool for identifying vulnerable groups during the COVID-19 pandemic. Once identified, those groups can follow recommendations (Ornell et al., 2020) to reduce anxiety such as exercising regularly, getting plenty of sleep and avoiding excessive alcohol and drug use (Center for Disease Control and Prevention, 2020). Finally, paying attention to mental care during and after the new coronavirus pandemic can guide us in selecting the appropriate mental skills necessary for the post-pandemic world.

In our study, the CFA has demonstrated that the coronaphobia construct extends to the Brazilian population in a similar way it was demonstrated to populations outside the country (United States of America) where the CAS was created such as Turkey (Evren et al., 2020), Bangladesh (Ahmed et al., 2020), Mexico (Mora-Magaña et al., 2020) and Peru (Franco-Jimenez, 2020). Interestingly, multigroup CFAs revealed that items #2 (sleep) and #3 (paralysis) were stronger indicators of coronavirus anxiety in women whereas appetite loss (item #4) was a stronger indicator of coronaphobia in younger adults (18 to 29 years old). These findings were not found in the original CAS investigation (Lee, 2020) but can be interpreted as important indicators of coronavirus anxiety in these two segments of the Brazilian population.

A ROC analysis demonstrated good diagnostic accuracy for CAS-BR and evidence of discrimination ability for functional impairment (AUC =.77, *p* < .001). Even though, Youden indices were all very low (<.50) and we were unable to identify the optimal cut-score for psychiatric screening purposes. The original CAS study (Lee, 2020) reported that CAS exhibited good diagnostic properties (AUC = 0.94, *p* < .001) and with an optimized cut score of ≥9 to classify adults as having dysfunctional anxiety (90% sensitivity and 85% specificity). This difference in sensitivity rates may rely on the fact that the Brazilian sample was characterized for having low CAS-BR scores. Two observations might have contributed to low CAS-BR scores in our sample. First, the data presented in this study was collected during a period that includes the peak and the beginning of the stabilization of cases per day of COVID-19 (Coronavírus Brasil, 2020). Since the number of cases and deaths started to stabilize, it is likely that people might have felt more hopeful. Second, it is possible that our sample was resilient to COVID-19 pandemic since it took a long time for the number of cases to peak in Brazil.

Although the aforementioned potential shortcomings within this research should be taken into consideration, bivariate correlation analyses strongly support the validity of the CAS-BR for measuring coronavirus anxiety. CAS-BR were shown to be positively correlated with scores from measures of functional impairment, generalized anxiety, and depression. Taken together, these results provide construct validity evidence for the CAS-BR. Furthermore, our data call attention to the fact that low- and middle-income countries are facing a serious mental health problem (Vigo et al., 2020).

Our study also revealed significant sociodemographic and background differences in CAS-BR scores. Specifically, the participants who were women, had a history of anxiety disorder, and participants very dissatisfied or dissatisfied with the Government’s responses to COVID-19 had higher CAS-BR scores compared to their counterparts.

The findings that women and participants with a history of anxiety exhibited higher CAS-BR scores compared to their counterparts were previously demonstrated in the original CAS study (Lee, 2020). Furthermore, studies conducted in Brazil (Abad et al., 2020; Barros et al., 2020) and other countries (Jia et al., 2020; Wang et al., 2020) suggested that women and participants with a history of anxiety are segments within the Brazilian population that are vulnerable to clinical anxiety. It is also argued that the greatest vulnerability of women to the stressors of the COVID-19 pandemic is related to socio-cultural factors (Campos et al., 2020). Interestingly, the CAS-BR items #2 (sleep) and #3 (paralysis) are stronger indicators of coronavirus anxiety for women. These findings are in line with a previous observation that women are more susceptible to experience sleep disorders (Barros et al., 2020).

The high coronavirus anxiety observed among participants very dissatisfied or dissatisfied with Government’s responses to COVID-19 might have reflected the lack of a plan or even national guidelines to face the pandemic. Brazil is one of the most affected countries by the pandemic in total numbers of cases and deaths (WHO, 2020). Many Brazilian political leaders, including the president, Jair Messias Bolsonaro, did not follow general WHO recommendations for delaying the virus propagation such as wearing masks and avoiding agglomerations. There was no plan by the federal government to reorient the economy in order to stimulate the necessary service and product sectors. The social support actions that would allow adherence to social isolation strategy were also timid (Henriques & Vasconcelos, 2020). An interesting sutdy showed that prolonged exposure to the pandemic news is directly related to the chance of developing mental disorders (Campos et al., 2020). It is important to note that the volume of inaccurate and false information disseminated in Brazil is high, generating feelings of insecurity, which can significantly increase the risk of developing psychological distress (Duarte et al., 2020). Thus, it is not surprising that individuals dissatisfied with the government had higher CAS-BR scores.

Our data also showed that individuals with a previous history of anxiety disorders are demographic segments of greatest vulnerability and are likely to be vulnerable groups during and after the pandemic. Therefore, it is necessary to guide these segments of the population to adopt practices to improve quality of sleep, to avoid long and unnecessary exposure to information published in the media, to practice of physical exercises, to sleep better, and to avoid consumption of alcohol and tobacco. These practices could reduce psychological distress in individuals with a previous history of anxiety disorders during and after the pandemic.

It is worth noting that this study has some limitations. First, this study was conducted online and potential participants without Internet access were not included in our sample. Second, the results of this study may be an underestimate of the psychological impact of the COVID-19 pandemic because the data were collected in a period that Brazilians might have already built some resilience to the crisis caused by the COVID-19 pandemic. Third, the results of this study may not generalize to all regions of Brazil because the sample used was mostly based on data (86.2%) collected in the south and southeast regions of Brazil. Fourth, the study lacks the sensitivity and specificity of the CAS-BR in detecting dysfunctional coronavirus anxiety. Fith, measures of mental health indicators were not carried out. Despite these limitations our findings are largely consistent with the results of previous investigations and support the validity of the CAS-BR for measuring coronavirus anxiety in Brazilian adults.

## Conclusion

In conclusion, our results can help to identify trends among people emotionally affected by the pandemic, particularly vulnerable groups such as women and people with history of anxiety disorder. Since some demographic segments are at greater vulnerability, these findings, collectively with other reports, could be of significant importance in guiding mental health policies.

In this context, the CAS-BR should be considered as a potential tool to assess the effectiveness of intervention programs for the management of anxiety during and after the COVID-19 pandemic.
